# Connexins and pannexins in neuronal development and adult neurogenesis

**DOI:** 10.1186/s12860-016-0089-5

**Published:** 2016-05-24

**Authors:** Leigh Anne Swayne, Steffany A. L. Bennett

**Affiliations:** Division of Medical Sciences, University of Victoria, Medical Sciences Building Rm 224, 3800 Finnerty Rd, Victoria, BC V8P5C2 Canada; Department of Biochemistry, Microbiology and Immunology, Neural Regeneration Laboratory, Ottawa Institute of Systems Biology, University of Ottawa, Ottawa, ON Canada

## Abstract

Connexins and pannexins share very similar structures and functions; they also exhibit overlapping expression in many stages of neuronal development. Here, we review evidence implicating connexin- and pannexin-mediated communication in the regulation of the birth and development of neurons, specifically Cx26, Cx30, Cx32, Cx36, Cx43, Cx45, Panx1, and Panx2. We begin by dissecting the involvement of these proteins in the generation and development of new neurons in the embryonic, postnatal, and adult brain. Next we briefly outline common mechanisms employed by both pannexins and connexins in these roles, including modulation of purinergic receptor signalling and signalling nexus functions. Throughout this review we highlight developing themes as well as important gaps in knowledge to be bridged.

## Background

**Fig. 1:**
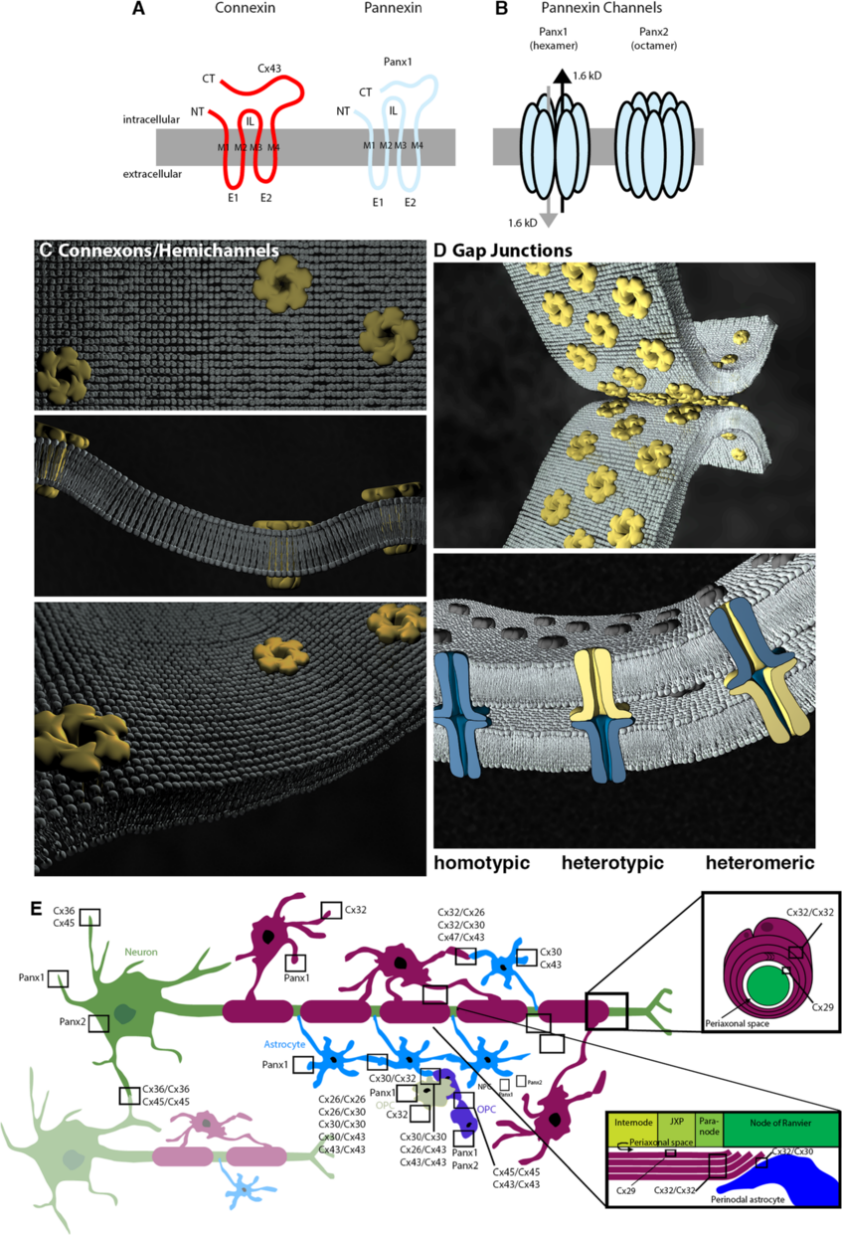
Cx and Panx nexuses. **a** Cxs are the structural units of single membrane channels (hemichannels) and intercellular channels (gap junctions). Panxs are primarily single-membrane channel proteins. Membrane topology in monomeric form (i.e., Panx1, blue) is strikingly similar to that of a connexin (i.e., Cx43, red). **b** Panxs exclusively form single-membrane pores composed of Panx1 hexamers, Panx2 octomers, or possibly Panx1/2 heterotypic channels of unknown numbers of protein subunits. **c** Cxs oligomerize to hexamers capable of forming single membrane channels (homotypic connexons or hemichannels are indicated). Schematic models crystal structure in a non-junctional lipid bilayer. **d** Axial alignment of compatible Cx connexons generate homotypic (blue connexon/blue connexon), heterotypic (yellow connexon/blue connexon), and heteromeric (blue-yellow connexon/blue-yellow connexon). **e** Cx26, Cx30, and Cx43 are expressed by astrocytes. Cx29, Cx32, and Cx47 are expressed by oligodendrocytes. Cx32 is expressed by oligodendrocyte precursor cells (OPCs). Cx36 and Cx45 are expressed by neurons. Cx45 and Cx30 are expressed by NPCs. Panx1 is expressed by astrocytes, OPCs, oligodendrocytes, and neurons. Panx2 is found in neurons and NPCs. Panx single membrane channels, Cx hemichannels/connexons, and Cx-compatible gap junction channels are depicted. Abbreviations: CT, carboxyl termini domain; E1/E2, extracellular loop domains; IL, intracellular loop; JXP, juxtaparanode; M1-M4, transmembrane domains; NT, amino termini domain. Representations are based on [125–127]

Connexins (Cxs) and pannexins (Panxs) are channel-forming proteins that play several important roles, both separate and over-lapping, in the regulation of neuronal development (for recent related reviews see [1, 2]). Both families help orchestrate complex arrays of cellular behaviours, including proliferation, migration, specification, and differentiation. While Cxs and Panxs share important structural similarities, critical differences indicate that their pore-forming functions are not redundant. For instance, despite both being defined by four transmembrane domains (with intracellular N- and C-termini) that oligomerize into higher order structures forming single membrane channels (Fig. 1a-c), Cxs and Panxs share no sequence homology at the protein level. Rather, their functional relationship is indirect from a genetic perspective and is somewhat historical in nature. Cxs are the structural subunits of both single membrane channels (also referred to as “hemichannels” or “connexons”) in non-junctional membranes (Fig. 1c) and gap junction channels (Fig. 1d). Axial alignment of two connexons creates (a) intercellular channels that directly connect the cytoplasm of adjacent cells and (b) reflexive channels that span adjacent membrane compartments, notably in myelinating oligodendrocytes (Fig. 1d, e). Panxs were initially identified through moderate sequence similarity to invertebrate gap junction forming proteins, the innexins [3, 4], however, the bulk of evidence suggests that endogenously expressed Panxs form primarily single membrane channels in non-junctional membranes and not intercellular channels [5] (Fig. 1b). Only compatible Cxs in the central nervous system (CNS) are capable of oligomerization, docking, hemichannel formation, and gap junctional intercellular communication (GJIC) [6] whereas both CNS Panxs, Panx1 and Panx2, can form homotypic hexamers (Panx1) or octomers (Panx2) and possibly heterotypic channels [7]. Thus, clustered assemblies of identical (homotypic Cxs or Panxs) or diverse (heterotypic Cxs and Panxs and heteromeric, Cxs only) pairings dictate network-specific permeabilities and gating properties and, in the case of the Cxs, participate in defining boundaries of cell-cell communication [6].

Despite these significant functional differences, Cxs and Panxs do share similar mechanisms in directing the development of neurons in both embryonic and adult brain. For example, Cx hemichannels and Panx single-membrane channels have both been implicated in mediating ATP release from neural progenitor cells (NPCs) and neighbouring, potentially “instructive”, neurons and glia cells (reviewed in [8, 9]). Critical autocrine and paracrine signalling pathways are triggered by the action of the released ATP on various types of ATP-sensitive (purinergic) receptors expressed by NPCs. Moreover, in adult tissues, cell-specific Cx and Panx expression and protein-protein-specific interactomes [10] define distinct neuro-glial networks implicated in the regulation of adult hippocampal neural progenitor cell fate [10–15]. However, between these similar single membrane channels, there remain important differences. For example, Cxs are sensitive to extracellular Ca^2+^, while Panxs are not [16]). The purpose of this review is to highlight some developing themes in this area, and to outline important gaps in knowledge in the growing body of work suggesting Cxs and Panxs play important roles in the development of neurons and associated cellular behaviours. Understanding these complexities has important implications for the understanding of and development of treatments for diseases of neurodevelopment and acquired brain injuries.

### The birth of new neurons occurs in the embryonic, postnatal and adult brain

The majority of neurons of the cerebral hemispheres are born during embryonic development from unspecialized cells collectively referred to as neural precursor cells (NPCs; reviewed in [17]). This is an umbrella term representing the spectrum of immature cells ranging from radial glia or stem like cells (having the potential to become any type of neural cell) to neuroblasts (committed to becoming neurons) that we will use throughout this review for simplicity. In the embryonic brain, cortical glutamatergic pyramidal neurons arise from ventricular zone (VZ) NPCs, while GABAergic neurons arise from NPCs in the ganglionic eminence. Developing neurons migrate to form the cortical layers and extend axons and dendrites outwards from the cell body as they differentiate. Axon and dendrite outgrowth as well as remodeling and development of synapses occurs during the early postnatal weeks (recently reviewed in [18]). Complex changes in cellular signalling and morphology are critical to facilitate the growth, development, and maturation of immature NPCs into neurons (recently reviewed in [19]).

**Fig. 2:**
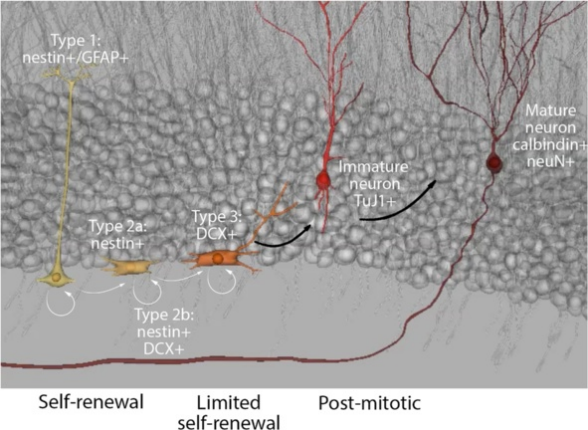
The adult SVZ and SGZ support neurogenesis. Adult NPCs are defined by their capacity to proliferate and replenish neuronal and glial numbers. Antigenic markers used to distinguish between lineages are listed

In the postnatal and mature brain, the subventricular zone (SVZ; a further specialization of the embryonic VZ) and, in the dentate gyrus of the hippocampus, the subgranular zone (SGZ) retain the ability to generate new neurons (Fig. 2), a process commonly referred to as postnatal, and/or adult neurogenesis (reviewed in [20]). Specification is a step-wise process. For example, in the SGZ, activated type 1 stem-like cells, identified by nestin and glial fibrillary acidic protein (GFAP) immunoreactivity, can generate nestin^+^/GFAP^−^ Type 2a progeny [21, 22]. Both type 1 and 2a populations are multipotential, although primarily restricted to a granule neuron phenotype in vivo [23, 24]. Neurogenesis is assured when Type 2a progenitors produce committed type 2b nestin^+^/doublecortin (DCX)^+^ cells that, in turn, specify to type 3 DCX^+^ neuroblasts before terminal specification. Type 3 neuroblasts migrate a short distance from the SGZ into the granule cell layer of the dentate gyrus where they can terminally differentiate into post-mitotic neurons [21, 22]. Some of these newly born neurons will integrate in dentate gyrus circuitry as granule neurons. The majority will be deleted prior to maturation [21, 25] (Fig. 2). In addition to primary cultures, many cell lines are also used in cell biological analysis of NPC cellular behaviours such as proliferation, migration, and neuritogenesis. The investigation of Cxs and Panxs in NPCs and developing neurons has spanned all of these models of NPC development: embryonic cortex, early postnatal and adult SVZ and hippocampal neurogenic niches, and cell line models.

### Cx and Panxs in NPCs in the embryonic, early postnatal and adult brain

Twenty mammalian Cxs have been identified in mouse; twenty-one in humans. Fourteen Cxs (Cx26, Cx29, Cx30, Cx30.2, Cx31.1, Cx31.9, Cx32, Cx36, Cx37, Cx40, Cx43, Cx45, Cx47, Cx57) and two Panxs (Panx1 and 2) are expressed in murine and human CNS [6, 26–30] (Here the murine Cx naming nomenclature is used.). The global expression pattern of at least nine of these Cxs (Cx26, Cx29, Cx31.1, Cx32, Cx36, Cx37, Cx43, Cx45, Cx47) and both Panxs changes over the course of cerebral development [31–38]. These changes correspond closely with the spatial-temporal patterns of embryonic and early postnatal neurogenesis and gliogenesis [31–35] and are recapitulated over the course of NPC differentiation in vitro [12, 13, 39–41]. Ray et al., [38] showed that Panx1 expression in neural crest-like cells is downregulated when cells adopt a Schwann cell-like glial lineage suggesting a role for Panx expression in gliogenesis. In postnatal hippocampus, we found that Panx2 localizes to subsets of multipotential NPCs both in vitro and in vivo and is transiently downregulated when cells commit to a neuronal lineage [11]. Panx1 also promotes NPC proliferation [42] as well as cell migration, while inhibiting neurite outgrowth [43] in vitro.

#### Embryonic neurogenesis

There are at least three ways in which Cx expression impacts upon embryonic neurogenesis. First, Cx-mediated GJIC between NPCs enables clusters of coupled cells to coordinate their responses to extrinsic stimuli. In the embryonic CNS, spatial-temporal patterns of synchronous cellular activity are observed between cells destined to become functional domains [44–46]. GJIC is recognized as one of the underlying mechanisms regulating this type of synchrony [47–53]. For example, proliferating units in the embryonic VZ are segregated from migrating units in the overlying cortex by engagement of α1β6 integrin receptors with laminins [54, 55]. We have shown that laminin differentially regulates Cx mRNA and protein expression [12]. Within these ECM-defined boundaries, GJIC ensures the creation of functional domains by enabling coordination between “like-lineage cells” yet isolation from neighbouring groups of cells induced by competing stimuli to specify to a different fate (reviewed in [56]). In addition to synchronous activity, Cx-mediated intercellular communication also permits NPCs to exchange small signalling molecules with adjacent “instructive” cells. In vitro, NPCs are directed to adopt a neuronal or oligodendrocytic lineage by juxtacrine communication with different populations of terminally differentiated feeder layers [24] presumably mediated, in part, by GJIC.

Regionalization is further influenced by the changing repertoire of Cxs expressed by adjacent populations over time. For example, Cx36, a neuronal specific Cx, is dynamically expressed over the course of embryonic neurogenesis [31, 57, 58] . Cx36 and, to a lesser extent, Cx26 localizes to the VZ during the first wave of neurogenesis [59]. Cx36 expression is dramatically reduced over the course of post-natal maturation, but remains enriched in subsets of interneurons in the hippocampus, olfactory bulb and thalamus, and is sparsely expressed in the neocortex [60–62]. In embryonic striatal derived murine NPC cultures, Cx36 is a positive regulator of neuronal differentiation [57]. While reports on Cx36 mRNA expression in embryonic brain in mouse [31, 58] and *Danio rerio* [63] note that expression overlaps with the period of neural induction, the authors did not specifically investigate Cx36 expression in NPCs making it likely Cx36 is expressed towards the end of neurogenic specification (although this hypothesis requires empirical validation).

The anticipated changes in Cx-mediated intercellular communication associated with these changes in protein expression, in addition to changes in Cx-mediated hemichannel formation and Panx-mediated single membrane pores in non-junctional membranes, are believed to play a role in coordinating activity of NPCs influencing NPC activation, tissue differentiation, NPC migration, regional specification, axonal growth and guidance, and synaptogenesis during CNS development [56, 64–66]. Functional indices of both electrical and metabolic coupling are widespread in the developing nervous system and thus gap junctions represent the predominant means of cell-cell communication between NPCs prior to formation of chemical synapses [45, 67]. Both electrical and metabolic coupling have been implicated in terminal regional specialization and the establishment of cortical circuits [53, 68]. Prenatal GJIC is thought to be involved in regulating the formation and stabilization of cortical neuronal circuits providing a means by which NPCs and newly born neurons can communicate until sufficiently mature to express neuron-specific neurotransmitters [32].

Second, the passage of ions and lipid second messengers through Cx-hemichannels in non-junctional membranes propagates sequential signalling waves to adjacent cells over great distances. During corticogenesis, hemichannel-mediated Ca^2+^ waves increase in number, amplitude, and distance travelled over the course of embryonic development [66]. Moreover, in the early postnatal neocortex, dendritic gap junctions mediate the propagation of inositol trisphosphate (IP3) and calcium waves. This process is thought to form the basis of functional regionalization by dividing the immature neocortex into columnar patches of coordinated activity [44, 69–71].

**Fig. 3:**
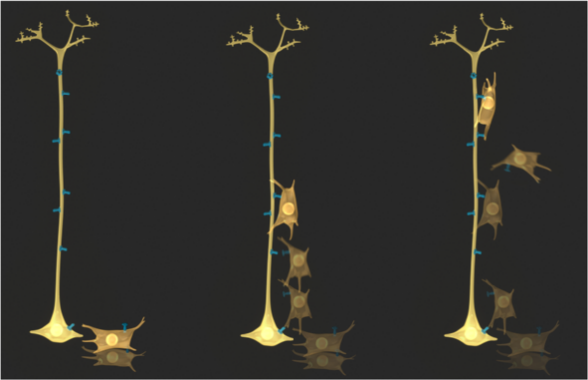
Connexon-Connexon mediated adhesion domains. In addition to forming functional intercellular channels, docking of compatible connexons between radial glia (yellow) and NPCs (gold) directs migration of NPCs Docking and undocking enables the “rolling” of NPCs along their radial glial guides to their final location before terminal differentiation. Adhesion can be channel-independent without requiring exchange of small molecules or functional channel opening

Finally, Cxs also mediate protein-protein interactions that affect regulation of NPC cell behaviours such as migration. These have commonly been referred to as ‘channel independent’ functions. The prime example in the embryonic brain is the adhesive properties of Cx43 allowing transient cell-cell interactions that direct cell migration without apparent formation of functional channels (Fig. 3) [72–74]. It is not completely clear whether (or which) protein-protein interactions are involved in other functions of Cx43 during cortical development such as the regulation of proliferation [75] and differentiation [76–78]. Cx43 interactions regulating cell proliferation have, however, been described in other cell types. For example, in human hepatocarcinoma cells, Cx43/Hsc70 interactions regulate the G1/S cell cycle check point [79]. Here, Cx43 sequesters Hsc70 at the plasma membrane preventing its interaction with the Cdk inhibitor p27 and therefore nuclear translocation of the cyclin D1-CDK4-p27 complex regulating transition through G1/S. Thus, increasing Cx43 levels inhibits cell proliferation. Inhibition can be rescued by Hsc70 overexpression. This example illustrates the idea that cell specific differences in Cx/Panx interacting proteins, in addition to expression levels of Cxs/Panxs themselves, has functional consequences. In another example, Cx43 has also been shown to interact with Ask1, an upstream activator of c-jun N-terminal kinase (JNK [80]) This interaction, in part, protects both C6 glioma cells and primary astrocytes from apoptosis induced by oxidative stress. Interesting, upregulation of Ask1 following inflammation also promotes neuronal differentiation [81]. As multipotential NPCs express Cx43, it would be important to test whether Ask1/Cx43 interaction enhances NPC survival and neurogenesis in the context of inflammation/pathology. Finally, the C-terminus of Cx43 has been shown to be a negative modulator of neuronal differentiation [77]. It has been suggested that the Cx43 C-terminus engages in important protein-protein interactions underlying this effect, however, the specific protein interaction partners involved have yet to identified. Moreover, while differentiation does not appear to be dependent on channel function [77, 78] (but see also [82, 83]), work by Cheng et al. [75] suggests that the regulation of proliferation by Cx43 does depend on channel function. Because these critical behaviours in the development of neurons: migration, proliferation and differentiation, often overlap and require very fine tuned transitions, there is likely some cross-talk between channel function and protein-protein interactions. It would be reasonable to speculate that conformational changes associated with changes in channel activity could impact on interactions with intracellular proteins. This underlies the impact of many types of ion channels and receptors on cell biology (for example see [84]). As new technologies reveal the finer details of the relationships between these, the term ‘channel independent’ may need to be revised.

#### Postnatal/adult neurogenic niches

The current state of knowledge of reported Cx and Panx expression patterns in different NPC populations is summarized in Fig. 1e and Fig. 2 with focus on the hippocampus. NPCs and immature neurons of the early postnatal and adult SVZ express Cx26 [85] Cx43 [85–91], Cx45 [85–87, 92] and Panx1 [42, 43, 85]. Dye coupling experiments suggested that NPCs (specifically radial glia) are connected with one another and with astrocytes [85, 87, 88, 90] or microglia [85] via gap junctions. Interestingly, the rare radial glia that are retained in postnatal retina also express Cx30 [93] suggesting a role for this Cx in the control of adult multipotential glial NPC fate. Lacar et al. [88] observed bidirectional Ca^2+^ waves travelling between NPCs and resident astrocytes via gap junctions. The Cx composition of these intercellular junctions was proposed to be Cx43 or Cx45 based on expression data, but this has yet to be experimentally tested using knockout (KO) mice and it is likely that Cx30 may also play a role given its localization in postnatal NPCs cultured in vitro and retina [12, 93]. Notably, Cx43 expression levels in SVZ NPCs increase with postnatal age [91] and inversely correlate with bromo-deoxyuridine labeling, an indicator of cell proliferation, suggesting that Cx43 negatively regulates cell proliferation (in contrast with its role in promoting proliferation during embryonic development [75]). Conversely, Cx45 was recently reported to promote NPC proliferation [92], in part through activation of purinergic receptors. Panx1 was also reported to promote proliferation [42] as well as cell migration, while inhibiting neurite outgrowth [43] in vitro. Treatment with CBX [86], which affects function of all four channel proteins (Cx30, Cx43, Cx45, Panx1), reduced migration of NPCs within the SVZ and rostral migratory stream (route of NPC migration towards the olfactory bulb).

In terms of the postnatal hippocampus, Rozental et al. [39–41] and Imbeault et al. [12] exhaustively characterized the repertoire of Cx expression therein. Cx26, Cx30, Cx37, Cx40, Cx43, and Cx45 mRNA and protein were expressed in various NPC populations. Using primary cultures of hippocampal NPCs, the authors demonstrated that expression changes dynamically over the course of neuronal commitment [39–41] and, as indicated above, that engagement with laminin differentially altered Cx expression in distinct NPC and oligodendrocyte precursor cell (OPC) populations [12]. As expected, Cx36 was also expressed in immature neurons. Kunze et al. [94] confirmed expression of Cx26, 30 and 43 in hippocampal NPCs in vivo, determining that deletion of Cx30 and Cx43 hampered proliferation of hippocampal NPCs. This is consistent with the role of Cx43 in promoting VZ proliferation in the embryonic brain [75], but seemingly contrasts with the association between high levels of Cx43 and reduced proliferation of SVZ NPCs in the postnatal/adult brain [91]. With respect to Panxs in the postnatal hippocampus, the highest expression levels of Panx2 in the hippocampus coincided with the period of peak postnatal hippocampus neurogenesis [11]. This corresponded with detection of Panx2 in several NPC populations. While Panx1 mRNA and protein was detected in primary postnatal hippocampal NPC cultures [42], its expression and role in hippocampal NPCs in vivo has not yet been determined.

#### Probing Cx and Panx function and spatiotemporal changes in expression using NPC cell lines

In order to address specific cell biological research questions as well as to bridge mouse to human studies, several cell lines have also been used to study the role of Cxs and Panxs in neuronal development. Cell line studies facilitated certain investigations such as expression analyses (over the course of differentiation) and siRNA knockdown, which were difficult to perform in situ due to the rarity and complexity of NPCs in the brain. For this reason, popular murine cell lines include Neuro2a (N2a) cells (derived from a murine neuroblastoma), P19 cells (derived from an murine embryonal carcinoma), NT2/D1 cells (derived from a human teratocarcinoma) and PC12 cells (an rat adrenal pheochromacytoma cell line) have been used to study Cxs and Panxs, primarily in the context of proliferation and differentiation. For example, Wicki-Stordeur et al. [42, 43] came to similar conclusions about Panx1 function in both primary postnatal SVZ cultures and N2a cell models in terms of a positive role in proliferation and a strong inhibitory role in neurite outgrowth. Recent work performed in vivo by Wicki-Stordeur et al. [95] confirmed that Panx1 regulates SVZ maintenance, through mechanisms that have yet to be determined. Similarly, Panx2 negatively regulated neurite outgrowth and differentiation in N2a cells [11].

The role of Cx43 in neuronal proliferation and differentiation has been widely studied in “NPC-like” cell line models with somewhat conflicting results. Moorby and Patel [95] made similar observations with respect to Cx43 in N2a cells to those previously made in postnatal SVZ [91], in that its overexpression increased the doubling time, which suggested a reduction in proliferation. Studies employing PC12 [96] and P19 cells [83] as well as their analogous human counterpart NT2/D1 cells [82] suggested that Cx43 acted as a positive regulator of neurite outgrowth and differentiation. By contrast, work with P19 and NT2/D1 cells revealed spatial-temporal changes in Cx43 (down-regulation) that were suggestive of negative regulation of neuronal differentiation [13, 97, 98]. Moreover, studies using the human NPC line ReNcell VM197 derived from the embryonic ventral midbrain suggested that siRNA mediated down regulation of Cx43 impaired both proliferation and differentiation [99]. The differing results and interpretations garnered from these in vitro models point to the need for more comprehensive understanding of Cx involvement in signalling pathways regulating these behaviours, including the relationship between channel function and signalling pathway/protein-protein interaction involvement. Furthermore, now that new tools are available that can be used to specifically target NPCs in vivo, and since issues with antibody-specificity have been resolved or can be addressed using null-mutant mice, many of these in vitro findings can now be confirmed in situ.

### Common mechanisms between Cx and Panx function in neuronal development

While it is fairly generally accepted that Cxs regulate the migration of NPCs through directly modulating cell-cell adhesion, the role of Panx1 in migration and the roles of Panxs and Cxs and Panxs in proliferation and differentiation are less well understood at the mechanistic level. A handful of reasonable hypotheses have emerged that warrant further study. Two of these include modulating ATP-sensitive purinergic receptor signalling, and setting up key signalling nodes through protein-protein interactions with multiple components of specific signalling pathways.

#### Modulation of purinergic receptor signalling

A large body of evidence suggests that Cx hemichannels and Panxs are capable of releasing ATP into the extracellular space (reviewed in [100]). Notably, it has been well established that both ionotropic purinergic (P2X) and metabotropic purinergic (P2Y) ATP receptor activation shapes embryonic and postnatal/adult neurogenesis (reviewed in [8, 9]). For example, activation of metabotrobic P2Y1 receptors increases NPC proliferation and migration in the VZ [101–105]. These effects are dependent on a number of physiological factors, such as crosstalk with growth factor receptor signalling [101, 105]. A similar mechanism has been identified in the adult hippocampus [106]. NPCs also express a variety of ionotropic P2X receptors over the course of embryonic and postnatal development (reviewed in [107]). For example, ionotropic P2X7 receptors are expressed in the embryonic VZ [108] and adult SVZ [109] and participate in the regulation of differentiation at all developmental stages. Downregulation of P2X7 receptors coincides with neuronal differentiation, and their inhibition stimulates differentiation and neurite outgrowth [110–112], suggesting that in some contexts, P2X7 receptors negatively regulate neuronal differentiation. However, in the embryonic brain, P2X7 receptors promote differentiation of NPCs [108]. Notably, P2X7 receptors are also involved in apoptosis [109, 113] and in the clearance of apoptotic cells [114], both of which are important in the regulation of neurogenesis and development of the brain. It has been suggested that expression of different P2X7 receptor isoforms could underlie these divergent actions of P2X7 receptors (reviewed in [115]). Moreover, the differential expression of multiple P2Y and P2X isoforms over the course of development (reviewed in [107]) adds several layers of complexity in terms of nucleotide signalling and the potential for crosstalk with Cx hemichannels and Panxs.

#### Cxs and Panxs as potential signalling nexuses in the brain

The term signalling ‘nexus’ (or node) refers to the capacity for ion channels and receptors (or any protein) to compartmentalize the components of one or more signalling pathways into close proximity to ion fluxes (or any signalling molecule or event) by virtue of providing interaction sites for multiple components of the pathway to enhance efficiency and speed of signalling (for discussion of examples see [84]). As described above, although this is related to the idea of ‘channel-independent’ functions, there is likely crosstalk between channel function and protein interactions, as occurs with many other ion channels and receptors. This role is likely very important in neuronal development, as multiple signalling pathways must be activated and deactivated in a highly orchestrated and strict manner for neuronal development to occur normally, as even subtle aberrancies in signalling during neuronal development, both embryonically and postnatally can lead to significant dysfunction, such as learning disabilities, autism, schizophrenia and epilepsies (for recent reviews see [116–118]). Cxs have emerged as important focal points for the organization of signalling systems (reviewed in [119–121]), but whether and how this occurs during neuronal development has yet to be elucidated. Recent work suggests that Panxs also likely form important signalling nodes ([43] and reviewed in [119, 122, 123]). At least initially, there appear to be some similarities between Cx and Panx interactomes. Both Cxs and Panxs have been shown to interact with multiple cytoskeletal components and modulators/regulators (for reviews see [119, 120, 123]). For example, in addition to interacting with actin, both Panx1 and Cx43 interact with actin-regulating proteins. Panx1 interacts with the actin-regulating protein Arp2/3 [43] and Cx43 interacts with Drebrin [124]. Interactions with both structural and functional components of the dynamic actin cytoskeleton could underlie their shared regulation of cell behaviours that require complex, concerted rearrangements of the actin cytoskeleton like neurite outgrowth and cell migration. The elucidation of the Cx and Panx interactomes over the course of neuronal development therefore represent a key area for bridging our current gaps in knowledge with respect to the role(s) of these proteins in regulating neuronal and brain development.

## Conclusions

Cxs and Panxs undoubtedly play important roles in neuronal development in the embryonic, postnatal, and adult brain. From this body of work, it has emerged that there is a great deal of expression and functional overlap between Cxs and Panxs in the context of neuronal development. Moving forward, dissecting the precise timing and roles of different Cxs and Panxs is now becoming more feasible due to the increasing availability of deleting Cxs and Panxs with greater cell type specificity and temporal precision. In addition to improved genetic manipulation (and improved validation), more potent, selective drugs for Cxs and Panxs and their signalling partners, such as the purinergic receptors will also improve our ability to dissect their combinatorial contributions. Furthermore, elaboration and functional study of Cx and Panx signalling nexuses will also provide greater understanding of the roles of these proteins in the proper development of the brain, and in disorders of neurodevelopment.
